# Perspectives on Chimeric Antigen Receptor T-Cell Immunotherapy for Solid Tumors

**DOI:** 10.3389/fimmu.2018.01104

**Published:** 2018-05-22

**Authors:** Paris Kosti, John Maher, James N. Arnold

**Affiliations:** ^1^Faculty of Life Sciences and Medicine, School of Cancer and Pharmaceutical Sciences, King’s College London, Guy’s Hospital, London, United Kingdom; ^2^Department of Immunology, Eastbourne Hospital, Eastbourne, East Sussex, United Kingdom; ^3^Department of Clinical Immunology and Allergy, King’s College Hospital NHS Foundation Trust, London, United Kingdom

**Keywords:** chimeric antigen receptor, T-cells, immunotherapy, tumor microenvironment, solid tumor

## Abstract

Chimeric antigen receptor (CAR) T-cell therapy entails the genetic engineering of a patient’s T-cells to express membrane spanning fusion receptors with defined specificities for tumor-associated antigens. These CARs are capable of eliciting robust T-cell activation to initiate killing of the target tumor cells. This therapeutic approach has produced unprecedented clinical outcomes in the treatment of “liquid” hematologic cancers, but to date has not produced comparable responses in targeting solid malignancies. Advances in our understanding of the immunobiology of solid tumors have highlighted several hurdles which currently hinder the efficacy of this therapy. These barriers include the insufficient accumulation of CAR T-cells in the tumor due to poor trafficking or physical exclusion and the exposure of infiltrating CAR T-cells to a panoply of immune suppressive checkpoint molecules, cytokines, and metabolic stresses that are not conducive to efficient immune reactions and can thereby render these cells anergic, exhausted, or apoptotic. This mini-review summarizes these hurdles and describes some recent approaches and innovations to genetically re-engineer CAR T-cells to counter inhibitory influences found in the tumor microenvironment. Novel immunotherapy drug combinations to potentiate the activity of CAR T-cells are also discussed. As our understanding of the immune landscape of tumors improves and our repertoire of immunotherapeutic drugs expands, it is envisaged that the efficacy of CAR T-cells against solid tumors might be potentiated using combination therapies, which it is hoped may lead to meaningful improvements in clinical outcome for patients with refractory solid malignancies.

## Introduction

Chimeric antigen receptor (CAR) T-cell immunotherapy reached a significant milestone in 2017, receiving its first approval by the U.S. Food and Drug Administration for two CD19-targeted CAR T-cells, Tisagenlecleucel (1) and Axicabtagene Ciloleucel (2). This achievement paves the way for further expansion of this therapeutic approach in the treatment of cancer. CAR T-cell therapy involves the isolation and *ex vivo* expansion of the patient’s peripheral blood T-cells, followed by genetic engineering of these cells to express CAR molecules on the cell surface, which have specificity for non-HLA-restricted tumor antigens. The genetically modified and expanded T-cells are then re-infused back into the patient, often following the administration of lymphodepleting chemotherapy (3).

**Figure 1:**
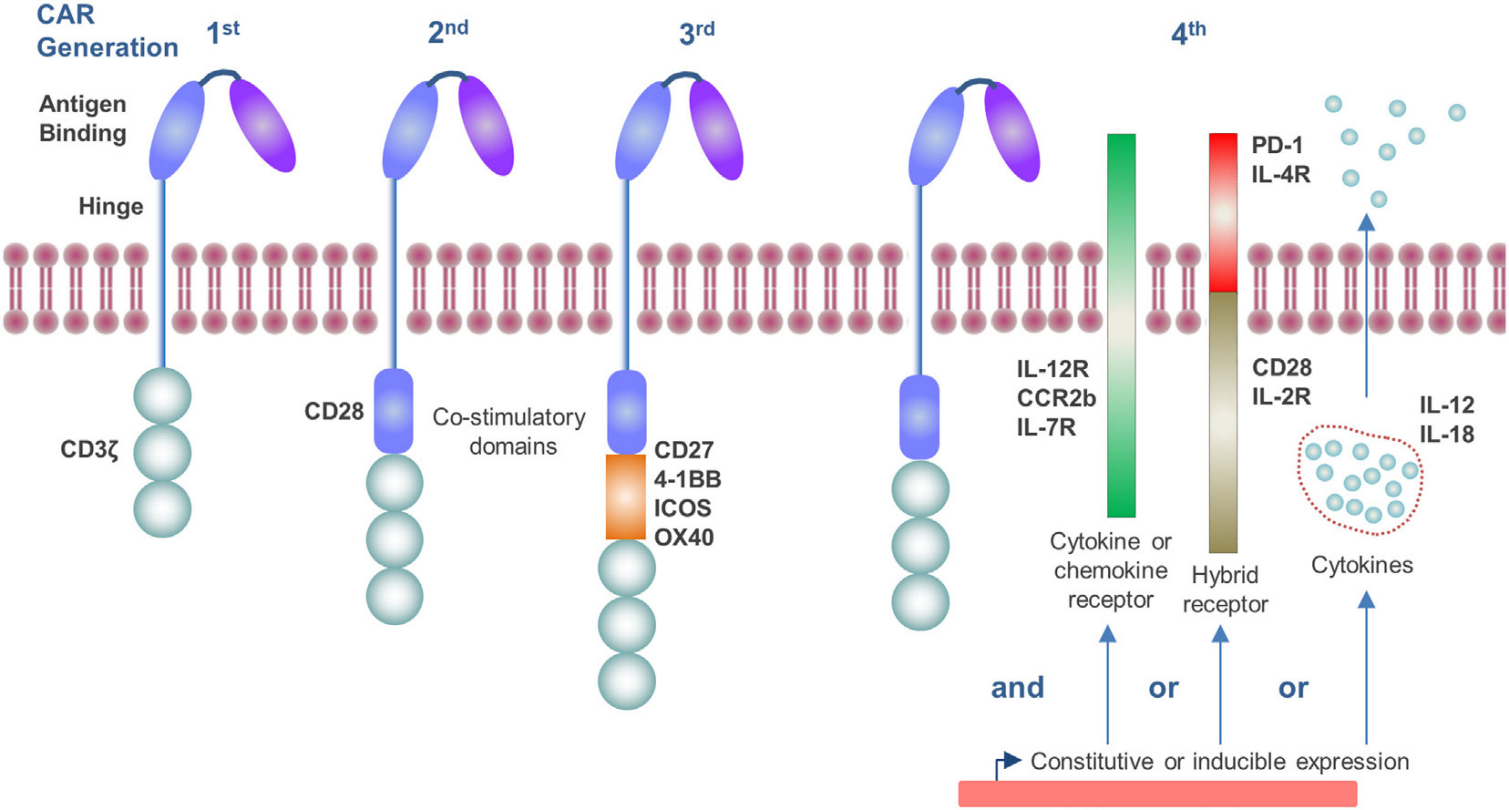
Generations of chimeric antigen receptor (CAR) molecules. First generation CARs contain a CD3ζ signaling endodomain. Second and third generation CARs, in addition to the CD3ζ domain, incorporate CD28 (second generation) or two or more additional co-stimulatory domains which may include CD27, 4-1BB, ICOS, or OX40 (third generation). Fourth generation CARs include constitutive or inducible expression of co-receptors or soluble cytokines alongside that of the CAR molecule which further promote T-cell activation.

The CAR construct has become progressively more sophisticated over time as our knowledge of T-cell activation and the tumor microenvironment (TME) has improved. The endodomain of CAR molecules, which transmits the activation signal from the ectodomain, contains a variety of signaling and co-stimulatory moieties which are indicative of their “generation” and can include CD3ζ, CD28, CD27, 4-1BB, ICOS, and OX40 (4, 5) (Figure 1). As such, CAR molecules circumvent the requirement to engage with exogenous co-stimulatory molecules for T-cell activation, which can be lacking in the TME and compromise CD8^+^ T-cell responses (6). More recently, CAR vectors have been designed to co-express auxiliary receptors and cytokines to improve T-cell function, which will be discussed later in this review (Figure 1).

Chimeric antigen receptor T-cell immunotherapy has achieved unprecedented clinical outcomes in patients with B-cell malignancies that previously had a very poor survival probability. At several centers, response rates consistently exceeding 80% have been reported in patients with relapsed/refractory B-cell acute lymphoblastic leukemia (ALL) (7–9) and lymphoma (10). Using anti-CD19 CAR T-cells in a Phase II trial involving 101 patients with B-cell lymphoma, 82% of patients had an overall objective response, and 54% had a complete response (2). Building on this highly impressive clinical data, CAR T-cells targeted against B-cell maturation antigen achieved a 89% overall response rate in 18 patients with evaluable multiple myeloma (11). Also, in a global multi-center Phase II trial, Tisagenlecleucel achieved an overall response rate of 81% in 75 pediatric and young adult patients with CD19^+^ relapsed or refractory B-cell ALL (12). With such impressive clinical responses, it is understandable that there has been significant interest in applying this therapy to solid malignancies, which account for the majority of cancer-related morbidity and mortality.

## Clinical Evaluation of CAR T-Cell Immunotherapy for Solid Tumors

Chimeric antigen receptor T-cells have been evaluated for the treatment of a variety of solid tumors (13–17). However, the proportion of patients responding with a measurable objective clinical response in these trials has been variable. Anti-disialoganglioside GD2 CAR T-cells have been used to treat evaluable pediatric patients with neuroblastoma, where 3 of 11 patients with active disease achieved complete remission (13, 18). However, in a trial using epidermal growth factor receptor-targeted CAR T-cells in patients with non-small cell lung cancer, partial disease remission in 2 of 11 patients was the best clinical response (15). There are also instances, using other CAR targets, where stable disease was the best clinical response (19, 20) or no objective clinical responses (21–23) have been detected. Although clinical responses of CAR T-cell therapy in solid tumors to date have not paralleled the success seen in liquid cancers [extensively reviewed (16)], the fact that clinical responses have been observed provides some encouragement.

## CAR Target Selection for Solid Tumors

A major hurdle in implementing CAR T-cell therapy against solid tumors is target selection. Since most solid tumors are of epithelial origin, the presence of tumor-specific antigens, which are absent on normal epithelial cells, is rare (24). This has resulted in instances of on-target off-tumor toxicities, such as was observed using Her2/neu targeted CAR T-cells in a patient with breast cancer (25). To expand the range of tumor-associated antigens (TAAs) that can be targeted, T-cell receptor (TCR)-mimetic CARs with specificity to HLA-presented antigens have been tested (26). CAR expression systems which exploit combinations of antigens, for example where ligation of a synthetic Notch receptor induces CAR expression (27), or tuning CAR affinity to preferentially target high density antigens (28), provide approaches to improve specificity. Dual-antigen targeting, where the CAR molecule can engage two separate TAAs can also be used to overcome antigen escape (29). Alternatively, the inclusion of inducible suicide switches which render CAR T-cells apoptotic have also been explored as a safety mechanism should toxicities arise (30, 31). The tumor stroma, which plays an important role in disease progression, has also been evaluated as a target. CAR T-cells targeting fibroblast activation protein alpha (FAP) which is expressed on the surface of cancer associated fibroblasts have shown efficacy in controlling tumor growth in preclinical models (32–34). However, as FAP^+^ stromal cells also play important roles in the periphery (35), off-tumor targeting of these populations by CAR T-cells results in cachexia and hematological toxicities in murine models, raising potential concern over FAP as a target (36).

Solid tumors contain various physical and environmental barriers which are not present in liquid cancers, and need to be considered. In the sections that follow, we consider the hurdles that T-cells encounter in the solid TME and how these may be overcome using novel innovations in CAR engineering or immunotherapeutic approaches.

## Physical and Environmental Barriers to CAR T-Cell Therapy in Solid Tumors

### Not Enough “Traffic” for CAR T-Cells

Unlike hematologic cancers where the infused CAR T-cells and tumor cells co-circulate in the blood, bone marrow, and lymphatics with ample opportunity for interaction, solid tumors represent discrete foci to which infused cells must migrate in order to contact the tumor cells and engage antigen. This involves chemotaxis in response to chemokines, such as CXCL-9, -10, and -11 (37). Since many human cancers display poor T-cell infiltration (“cold tumors”) (38), it is unsurprising that migration of CAR T-cells to the TME can be inefficient. Even the i.v. infusion of large numbers of CAR T-cells does not improve the clinical outcomes when tackling solid tumors, especially in patients with bulky disease; however, these trials were using the early first generation CARs (39, 40) (Figure 1). When clinically feasible, intra-tumoral injection has been demonstrated *in vivo* to circumvent poor trafficking of CAR T-cells, leading to tumor regression without systemic toxicity (41). In addition to the lack of T-cell chemokines being secreted by the TME, there are other chemokines, such as CXCL12, which actively inhibit T-cell migration into the tumor through engaging CXCR4 on their cell surface (42, 43). CXCL12 is highly expressed in variety of carcinomas, including pancreatic (44, 45), ovarian (46), and breast (47). However, pharmacologically blocking CXCR4 on the T-cell surface facilitated infiltration of T-cells into a spontaneous murine model of pancreatic ductal adenocarcinoma (43). As such, the CXCR4/CXCL12 axis may represent a therapeutic target to facilitate CAR T-cell infiltration in some solid tumors. Extravasation of T-cells into the tumor from the blood can also be inefficient due to the expression of molecules such as endothelin B receptor (ET_B_R) on the endothelium of the blood vessels within the tumor. High ET_B_R expression results in the nitric oxide-mediated deregulation of endothelial ICAM-1 expression, which reduces T-cell adhesion and compromises their ability to extravasate (48). Several attempts have been made to improve CAR T-cell trafficking to tumors (49–51). Kershaw and colleagues have demonstrated that expression of CXCR2 improved T-cell migration in a melanoma tumor which produced CXCL1, a chemokine commonly secreted by tumor cells (52). Similarly, CCR2b, the chemokine receptor for CCL2, has been co-expressed on CAR T-cells to exploit the CCR2/CCL2 axis, which facilitates myeloid cell recruitment into the tumor. CCR2b-expressing GD2-specific CAR T-cells had a 10-fold improvement in their migration to CCL2-producing neuroblastoma and demonstrated better *in vivo* antitumor activity in murine models (50). A similar observation was also achieved using mesothelin-directed CAR T-cells in murine models of mesothelioma (51). Others have improved CAR T-cell trafficking and the antitumor response *in vivo* using CAR T-cells that express a regulatory subunit I anchoring disrupter (RIAD) peptide that inhibits the protein kinase A-mediated suppression of the TCR, which can occur in the TME (53).

There are also physical barriers that restrict T-cell infiltration within solid tumors, where extracellular matrix proteins, such as proteoglycans and collagen, can restrict entry (54, 55). To overcome this, CAR T-cells have been engineered to express heparanase, which degrades heparin sulfate proteoglycans (56). This approach enhanced CAR T-cell infiltration within the tumor, leading to improved overall survival in xenograft tumor models.

### Immune Checkpoint Molecules

Intra-tumoral T-cells are often functionally tolerant, or suppressed, displaying reduced effector functions compared to peripheral T-cell pools, including lowered cytokine, perforin, and granzyme-B expression (57, 58). This phenomenon has also been observed for CAR T-cells (59). Two receptors that have been of particular interest in inducing this anergic T-cell state are PD-1 and CTLA-4, which represent part of a family of regulatory receptor known as checkpoint molecules, which prevent inappropriate immune activation, but can be exploited by cancer. For example the ligands for PD-1, PDL-1 and PDL-2, can be found expressed on a variety of tumor and stromal cells (60–62). Antibody-mediated blockade of immune checkpoint receptors that are expressed on the T-cell surface have shown unprecedented clinical activity in the treatment of solid tumors by sustaining endogenous antitumor immune responses, most notably in melanoma (63). The checkpoint molecules offer significant scope as therapeutic targets for combination therapy, as these molecules behave in a hierarchical structure within each TME (43, 61). Indeed, combined PD-1 and CTLA-4 blockade (using nivolumab and ipilimumab, respectively) has improved response rates in patients with advanced melanoma, compared to that of the single therapies (64). Importantly, regulatory molecules such as PDL-1 are upregulated by effector molecules of T-cell activation, such as interferon (IFN)-γ (60, 65), suggesting that the greater the T-cell activity, the more suppressive the TME becomes. Anti-HER2 CAR T-cell therapy has an enhanced antitumor response in preclinical murine models when combined with PD-1 blockade (66). The combination of immune checkpoint blockade with CAR T-cell immunotherapy is now under study in clinical trials (67). Innovations to CAR T-cells to permit insensitivity to immune checkpoints are also under investigation at the preclinical stage, involving such approaches as genetic inactivation of the PD-1 gene (68), co-expressing dominant-negative variants of the inhibitory phosphatases, such as Src homology 2 phosphatase (SHP-2), which mediate the signaling of checkpoint receptors (69), or the expression of PD-1 receptors with no signaling moiety as decoy molecules (70). Others have attempted to positively exploit the interaction by fusing the PD-1 ectodomain to the CD28 cytoplasmic tail to induce a co-stimulatory, instead of inhibitory, signal upon PDL-1 binding (71). Brentjens *et al*. enhanced the antitumor efficacy of CD19-specific CAR T-cell *in vivo* by co-expressing the activating checkpoint molecule, CD40-ligand (72). CD40L co-expression resulted in an increased CAR T-cell proliferation, Th1 cytokine secretion, and increased cytotoxicity *in vivo*. The stroma, as well as the tumor cells, play a fundamental role in suppressing T-cells responses in the TME. Our group has shown that tumor-associated macrophages (TAMs) can inhibit immune-mediated tumor rejection *in vivo* through their expression of the heme-degrading enzyme heme oxygenase-1 (HO-1) (61, 73). Carbon monoxide is one of the by-products of heme catabolism by HO-1 and has been demonstrated to be capable of suppressing T-cell proliferation, IL-2 secretion (74), and T-cell effector function (61, 75, 76). The pharmacological inhibition of this enzyme within the TME of a murine model of breast cancer resulted in a rapid restoration of a chemotherapy-elicited antitumor CD8^+^ T-cell response and immunological control of tumor growth, leading us to propose that HO-1 should also be considered as an immune checkpoint molecule (61). As HO-1 is expressed in a variety of cancers (77), it may warrant therapeutic targeting in CAR T-cell therapy. Together, these considerations present a strong rationale for combining CAR T-cell and immune checkpoint blockade therapies. Appropriate combinations of these therapies may improve CAR T-cell efficacy in the TME and support more favorable clinical response rates.

### The Anti-Inflammatory Cytokine Environment

The TME is a chronic inflammatory site and contains a variety of cytokines and chemokines, which influence the immune response. The TME is often skewed toward favoring pro-tumor Th2 cytokines, such as IL-4 and IL-13, rather than antitumor Th1 cytokines like IFN-γ and tumor necrosis factor-β (78). Cytotoxic T-cell function is depressed by cytokines, such as IL-4, IL-10, and transforming growth factor (TGF)-β, which are prevalent in the TME (79, 80). TAMs are an abundant stromal cell type in solid tumors and have been shown to secrete IL-10 as well as IL-6, which also suppresses T-cell cytolytic function and proliferation (81, 82). Similarly, regulatory T-cells (Tregs; CD4^+^ CD25^+^ FoxP3^+^) produce significant quantities of suppressive cytokines, including IL-10, TGF-β (83), and IL-35 (84). T-cells, which were engineered to express a dominant-negative form of the TGF-β receptor, have been demonstrated to have augmented antitumor responses in TGF-β producing tumors (85, 86). Alternatively, IL-4 signaling has been exploited to promote CAR T-cell expansion and activity. In one such approach, the IL-4 ectodomain has been fused to the common β chain of IL-2 and IL-15 receptors in order to achieve selective *ex vivo* amplification of CAR transduced T-cells. This approach renders it feasible to manufacture CAR T-cells from whole blood, circumventing the need for leukapheresis (87) and has the added benefit of augmenting CAR T-cell responses against tumors containing IL-4 in the TME (88, 89). Alternatively, the IL-4 receptor ectodomain was fused to the IL-7 receptor endodomain and co-expressed in prostate stem cell antigen-specific CAR T-cells in order to transduce a T-cell proliferation signal in response to IL-4 (90). The co-expression of this receptor led to improved *in vivo* CAR T-cell expansion and antitumor response. IL-12 is a potent inflammatory cytokine which enhances T-cell expansion and antitumor immune responses (91, 92). In murine preclinical tumor models, CAR T-cells which constitutively express IL-12 have been demonstrated to have enhanced proliferation, *in vivo* expansion, cytotoxicity, and antitumor efficacy (93–95). To circumvent off-target toxicities of this approach to expedite clinical translation, inducible IL-12 expression linked to CAR engagement has also been demonstrated to improve antitumor responses (96). CAR T-cells co-expressing IL-18 (97, 98), a constitutively active IL-7 cytokine receptor (99) or a tethered form of IL-15 (IL-15 peptide fused to IL-15Rα *via* flexible linker) (100) have also all augmented their antitumor response *in vivo*. It is clear that the cytokine environment within the TME is not conducive to permitting CD8^+^ T-cell activation and effector function. However, innovative ways to modulate and positively exploit the response of the CAR T-cells, such as through hybrid receptors, or IL-12 (Figure 1) show promising preclinical data and have started to progress through to clinical trials (NCT02498912 and NCT01818323).

### Metabolism-Associated Immune Suppression in the TME

Tumor cells are highly metabolically active with increased glycolysis and glutaminolysis (101). These metabolic pathways result in a distinct accumulation of metabolites in the TME which can compromise CAR T-cell function. Lactate is a metabolite derived from the glycolytic-pathway that is highly produced by tumor cells and directly suppresses proliferation, cytokine production, and effector function of human cytotoxic T lymphocytes (102). Prostaglandins, which are derived from prostaglandin E2 synthase and cyclooxygenase (COX)-1/2-mediated catabolism of arachidonic acid, can also suppress T-cell function (103). In keeping with this, aspirin (a COX inhibitor), has been demonstrated to potentiate immune checkpoint therapy in improving CD8^+^ T-cell responses (104) and may warrant investigation in combination with CAR T-cell immunotherapy.

A number of amino acid-degrading enzymes that are commonly expressed in the TME can also suppress T-cell function. These include indoleamine-2,3-dioxygenase (IDO) and tryptophan-2,3-dioxygenase (TDO) which degrade tryptophan, and arginase-1 and nitric oxide synthase (NOS) which degrade l-arginine. T-cells have been demonstrated to be particularly sensitive to the depletion of these amino acids, resulting in impaired proliferation and effector function (105–107) and increased T-cell apoptosis (108, 109). As well as the physical depletion of these amino acids, the catabolites of tryptophan degradation such as l-kynurenine and 3-hydroxyanthranilic acid have also been demonstrated to be immunosuppressive (110, 111). IDO inhibitors have been demonstrated to enhance CAR T-cell efficacy (111), and these are now being evaluated in clinical trials (112, 113). Arginase activity has also been demonstrated to inhibit proliferation and cytotoxity of GD2-specific CAR T-cells (114). The degradation of l-arginine by the NOS pathway also results in the generation of reactive nitrogen species (RNS). Myeloid-derived suppressor cells (Gr-1^+^ CD11b^+^ cells) and TAMs are potent sources of reactive oxygen species (ROS), which inhibit T-cell function (115). Both ROS and RNS are believed to induce T-cell tolerance by altering the flexibility of the TCR chains, which impairs the binding and responsiveness of CD8^+^ T-cells to peptide–MHC complexes (116). It would be interesting to consider how ROS/RNS modifications might also influence the antigen binding capabilities of the CAR. ROS/RNS may also inhibit T-cell infiltration into the tumor through inactivating CCL2 by nitration (117). Metabolites generated in the tumor, once regarded as by-products, are now accepted as crucial immune-modulatory molecules in their own right. As such, the metabolic pathways that facilitate immune-regulation may require therapeutic targeting alongside CAR T-cell therapy, such as IDO, which is currently under clinical investigation.

## Conclusion

**Figure 2:**
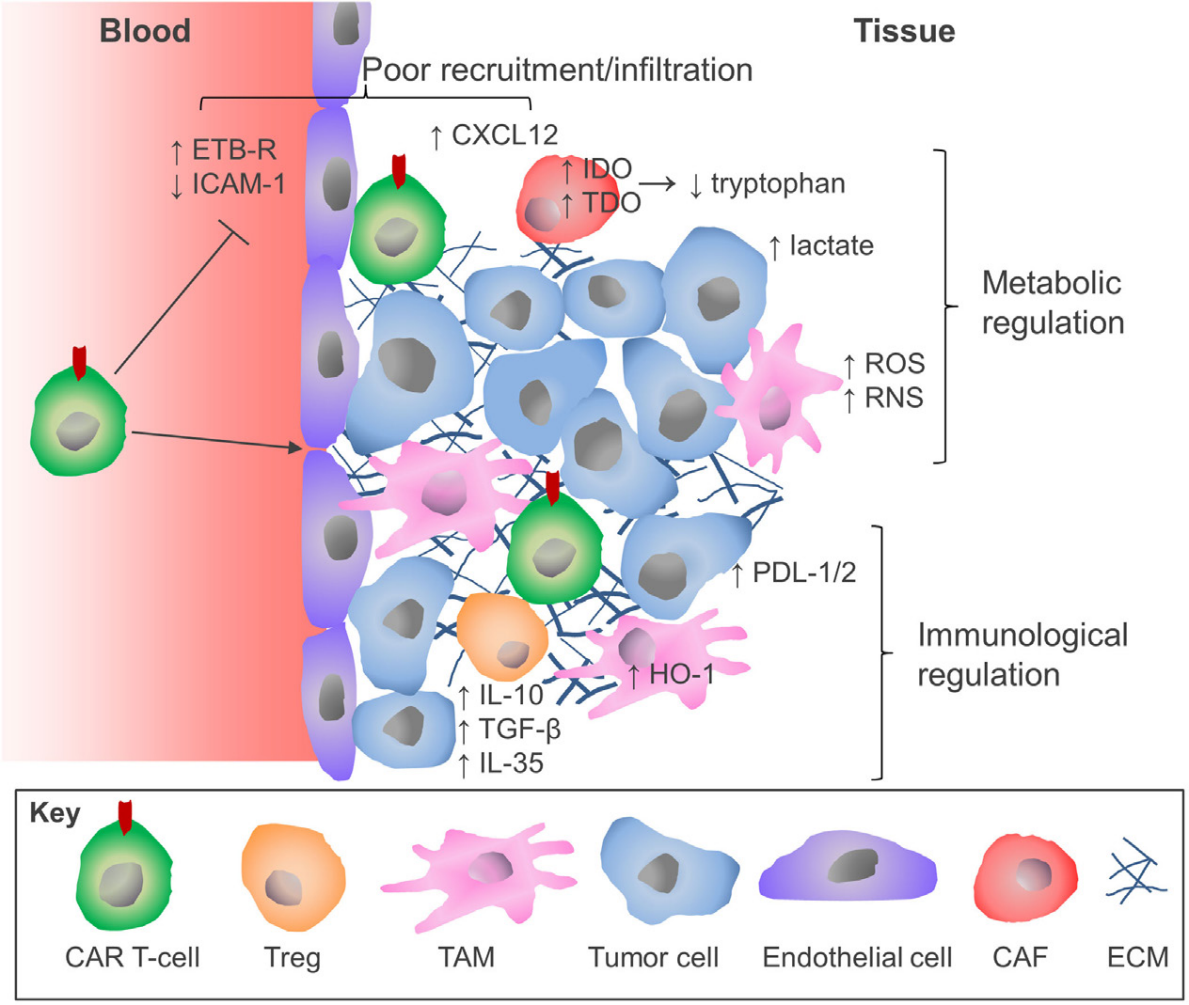
Overview of the barriers to CAR T-cells in solid tumors.

The success of CAR T-cell immunotherapy for hematological malignancies heralds a new era in the treatment of malignant disease. However, as this review has highlighted, the attainment of comparable clinical outcomes for patients with solid tumors will require considerable refinement of this therapeutic approach. Although there have been some encouraging recent case reports (118), CAR T-cells are subject to several additional constraints in patients with solid tumors which has hindered progress (Figure 2). The parallel success of immune checkpoint blockade therapies presents an opportunity for realignment of these distinct forms of immunotherapy through combinatorial therapeutic regimens. Similar pharmacologic opportunities are presented by the combination treatment with traditional cancer therapeutic modalities including radiotherapy (119) and chemotherapy (120), which have been demonstrated to sensitize tumors to CAR T-cell therapy, or molecular antagonists of inhibitory mechanisms that operate in the TME (e.g. IDO or HO-1 inhibitors). Equally, there are many opportunities for innovative re-engineering of CAR T-cells to deal with the physical and environmental barriers found in the solid TME. The configuration of these therapies represents the next challenge for the CAR T-cell field and will no doubt lead to meaningful improvements in the clinical response rates for the application of CAR T-cell therapy against solid malignancies.

## Author Contributions

All authors listed have made a substantial, direct, and intellectual contribution to the work and approved it for publication.

## Conflict of Interest Statement

The authors declare that the research was conducted in the absence of any commercial or financial relationships that could be construed as a potential conflict of interest.
